# Characterization of the innate immune response to chronic aspiration in a novel rodent model

**DOI:** 10.1186/1465-9921-8-87

**Published:** 2007-11-27

**Authors:** James Z Appel, Sean M Lee, Matthew G Hartwig, Bin Li, Chong-Chao Hsieh, Edward Cantu, Yonghan Yoon, Shu S Lin, William Parker, R Duane Davis

**Affiliations:** 1 Transplant Immunobiology Laboratory, Department of Surgery, Duke University Medical Center, Durham, NC 27710, USA; 2 Box 3864, Department of Thoracic Surgery, Duke University Medical Center, Durham, NC 27710, USA

## Abstract

**Background:**

Although chronic aspiration has been associated with several pulmonary diseases, the inflammatory response has not been characterized. A novel rodent model of chronic aspiration was therefore developed in order to investigate the resulting innate immune response in the lung.

**Methods:**

Gastric fluid or normal saline was instilled into the left lung of rats (n = 48) weekly for 4, 8, 12, or 16 weeks (n = 6 each group). Thereafter, bronchoalveolar lavage specimens were collected and cellular phenotypes and cytokine concentrations of IL-1alpha, IL-1beta, IL-2, IL-4, IL-6, IL-10, GM-CSF, IFN-gamma, TNF-alpha, and TGF-beta were determined.

**Results:**

Following the administration of gastric fluid but not normal saline, histologic specimens exhibited prominent evidence of giant cells, fibrosis, lymphocytic bronchiolitis, and obliterative bronchiolitis. Bronchoalveolar lavage specimens from the left (treated) lungs exhibited consistently higher macrophages and T cells with an increased CD4:CD8 T cell ratio after treatment with gastric fluid compared to normal saline. The concentrations of IL-1alpha, IL-1beta, IL-2, TNF-alpha and TGF-beta were increased in bronchoalveolar lavage specimens following gastric fluid aspiration compared to normal saline.

**Conclusion:**

This represents the first description of the pulmonary inflammatory response that results from chronic aspiration. Repetitive aspiration events can initiate an inflammatory response consisting of macrophages and T cells that is associated with increased TGF-beta, TNF-alpha, IL-1alpha, IL-1beta, IL-2 and fibrosis in the lung. Combined with the observation of gastric fluid-induced lymphocyitic bronchiolitis and obliterative bronchiolitis, these findings further support an association between chronic aspiration and pulmonary diseases, such as obliterative bronchiolitis, pulmonary fibrosis, and asthma.

## Background

Gastroesophageal reflux disease (GERD) has been associated with a number of pulmonary diseases, including idiopathic pulmonary fibrosis, asthma, chronic bronchitis, cystic fibrosis, and chronic obstructive pulmonary disease [1-5]. It is generally believed that GERD-associated pulmonary pathology is mediated by repetitive aspiration events. Indeed, GERD is said to be the most common cause of chronic intermittent aspiration [6,7]. DeMeester *et al*. found that 70% of patients with respiratory symptoms of persistent cough, wheezing, or recurrent pneumonia had GERD based on 24-hour pH monitoring of the distal esophagus [8].

It is likely that many of these pulmonary responses to repetitive aspiration are related to immune-mediated events. Lung transplant recipients with GERD represent one group for whom a chronic aspiration-induced immune reaction likely results in a particularly adverse clinical effect. Data from a number of retrospective clinical studies performed at our institution implicates chronic aspiration in the context of GERD as a reversible cause of pulmonary dysfunction and bronchiolitis obliterans syndrome (BOS) in lung transplant recipients [9-13]. The prevalence of pH-confirmed GERD is particularly high among patients with end-stage lung disease, approaching 50%, but increases to over 70% following lung transplantation [9,10,14]. However, lung transplant recipients with GERD that undergo antireflux surgery early in the posttransplant period exhibit decreased rates of BOS, mortality, and acute rejection [9,14].

Based on these data, our group has proposed that chronic aspiration associated with GERD may represent a repetitive non-allogeneic stimulus for immune-mediated injury in lung transplant recipients. Chronic aspiration may facilitate an innate immune response, predisposing lung transplant recipients to acute and chronic rejection. Furthermore, since these rejection processes are thought to involve primarily cell-mediated responses, it is possible that chronic aspiration additionally initiates or recruits an acquired immune response, facilitating or exacerbating pulmonary allograft dysfunction.

Although mounting evidence supports the clinical association between GERD and pulmonary dysfunction in lung transplant recipients and in patients with pulmonary fibrosis and end-stage lung disease, the physiologic and immunologic reaction to chronic aspiration has not been investigated using a suitable model system. In order to better characterize the changes in the lung that result from chronic aspiration, our laboratory has developed a rodent model of repetitive gastric fluid aspiration. Herein, we describe the inflammatory response to chronic aspiration in this novel rodent model. The results shed light on the mechanisms by which chronic aspiration may lead to pulmonary fibrosis, exacerbate end-stage lung disease, or stimulate an allogeneic response in lung transplant recipients.

## Methods

### Animals

Male, pathogen-free F344 rats were obtained from Charles River Laboratories (Wilmington, MA). All experiments were performed in accordance with the Guide for the Care and Use of Laboratory Animals prepared by the National Academy of Sciences and published by the National Institutes of Health. Protocols were approved by the Duke University Medical Center Institutional Animal Care and Use Committee.

### Collection of gastric fluid

Rats were anesthetized using inhaled isoflurane. A surgical gastrotomy was performed through which a silastic catheter was inserted. Gastric fluid was collected by gravity over a 12-hour period, the fluid from several animals was pooled together, and the mixture was then filtered through a 70-micron strainer before being stored at -80°C until immediately prior to use. The pH of all specimens utilized for experimentation was 1.0 – 2.5. Animals were not fasted prior to gastric fluid collection.

### Instillation of gastric fluid or normal saline

Male pathogen-free F344 rats (250–300 g) were sedated with ketamine (40 mg/kg IM), intubated orotracheally using the sheath from a 14-gauge IV catheter, and maintained on a mechanical ventilator (Inspira, Harvard Apparatus, Holliston, MA). Rats were subsequently disconnected from the ventilator and placed in the left lateral decubitus position with their head elevated at a 30° angle. A silastic catheter was inserted through the endotracheal tube to a distance 5 mm past the tip, where 150 microliters of either gastric fluid or normal saline (0.9% NaCl) was slowly injected. Rats were maintained in this position for 15 minutes and then extubated after recovering from sedation.

**Figure 1:**
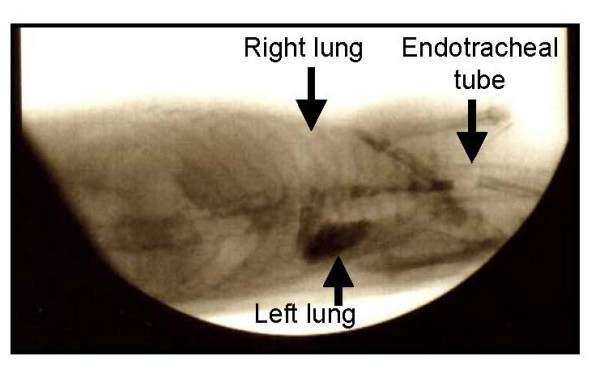
Instillation of fluid into the left lung. The instillation of gastrograffin into the distal trachea of sedated rats in the left lateral decubitus position resulted in consistent localization in the left lung, sparing the right.

Methods of the instillation procedure were developed based on studies in our laboratory demonstrating that gastrograffin contrast could be consistently instilled into the left lung while sparing the right lung with 100% reproducibility (Figure 1). The volume of fluid administered was equal to one half the lethal dose used in acute aspiration studies [15,16]. With the exception of control rats (n = 6), instillation of gastric fluid or normal saline into the left lung was repeated on a weekly basis for a predetermined period (i.e. 4, 8, 12, or 16 weeks, n = 6 in each group). A weekly regimen was selected after preliminary studies indicated that more frequent aspiration resulted in an unacceptably high mortality rate.

### Sample collection

One week after the predetermined number of left lung instillations, rats were sedated and a tracheotomy was performed through which rats were intubated. The trachea, lungs, and heart were then explanted en bloc. The right and left mainstem bronchi were sequentially lavaged with a total of 6 ml PBS buffer (37°C). 1 ml of bronchoalveolar lavage (BAL) specimens was centrifuged and the supernatant stored at -80°C for cytokine analysis. The remainder of the BAL specimens was utilized for FACS analysis.

### Histology

Lung tissue was fixed using 2% paraformaldehyde and stained using hematoxylin and eosin as well as Masson trichrome stain for collagen. The extent of fibrosis in trichrome-stained specimens was graded by a pathologist in a blinded fashion using a numerical scale described elsewhere [17].

### Flow cytometry

The phenotypes of cells in BAL specimens were determined using FITC-conjugated anti-rat CD172a (Serotec, Oxford, UK), TCR, CD4, and CD8 (Becton Dickinson, Franklin Lakes, NJ) monoclonal antibodies and quantified using a FACSCalibur flow cytometer (Becton Dickinson, Franklin Lakes, NJ).

### Cytokine assays

BAL specimens were thawed and concentrations of IL-1alpha, IL-1beta, IL-2, IL-4, IL-6, IL-10, GM-CSF, IFN-gamma, and TNF-alpha were measured using Bio-Plex multiplex bead-based immunoassays (BioRad Laboratories, Hercules, CA). Specimens were analyzed using a Luminex 100 flow-based, dual-laser array reader (Luminex, Austin, TX) and concentrations quantified using Bio-Plex Manager Software (BioRad Laboratories, Hercules, CA). TGF-beta concentrations were determined using ELISA-based sandwich immunoassays (R&D Systems, Minneapolis, MN).

### Statistical analysis

Unless otherwise noted, reported values represent mean ± standard error of the mean. Chi-square, ANOVA, and unpaired Student's t-tests were performed, where appropriate. For all statistical calculations, a p-value < 0.05 was considered significant.

## Results

### Histology

**Figure 2:**
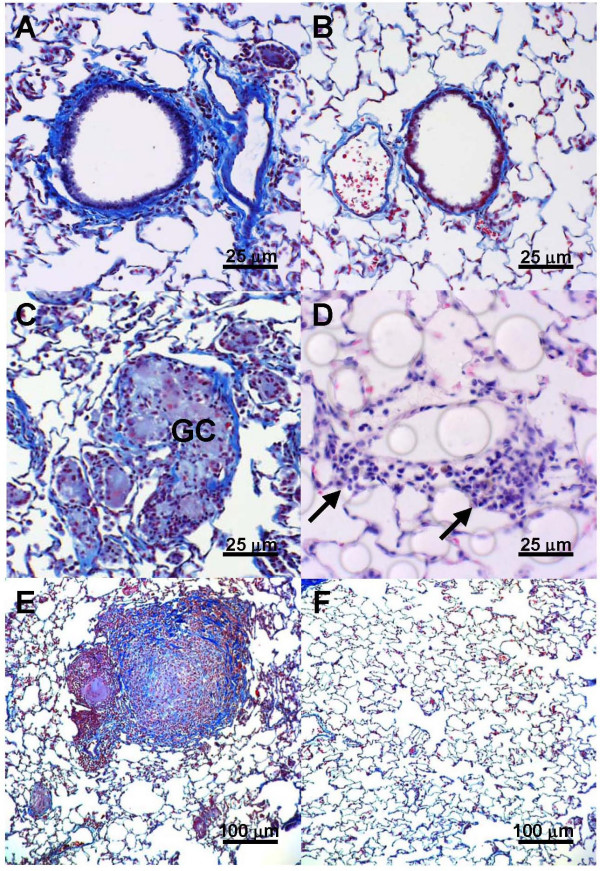
Histology following chronic gastric fluid aspiration. Evaluation of Masson trichrome-stained tissue demonstrated an increase in peribronchiolar and interstitial fibrosis in (a) left lung specimens after 8 weeks of gastric fluid aspiration compared to (b) lung specimens from untreated rats or rats receiving normal saline. Scattered cellular infiltrates were most apparent in left lungs following chronic aspiration of gastric fluid after 8 weeks and consisted primarily of (c) giant cells (GC), apparent in specimens stained with trichrome and (d) perivascular lymphocytes (arrows) as noted in specimens stained with H&E. In many trichrome-stained left lung specimens from rats receiving gastric fluid, (e) complete airway occlusion was observed reminiscent of lesions observed in lung transplant recipients exhibiting obliterative bronchiolitis (OB). Neither fibrosis nor cellular infiltrates were apparent in right lung specimens from rats undergoing gastric fluid aspiration for (f) 8 weeks or at other time points.

**Figure 3:**
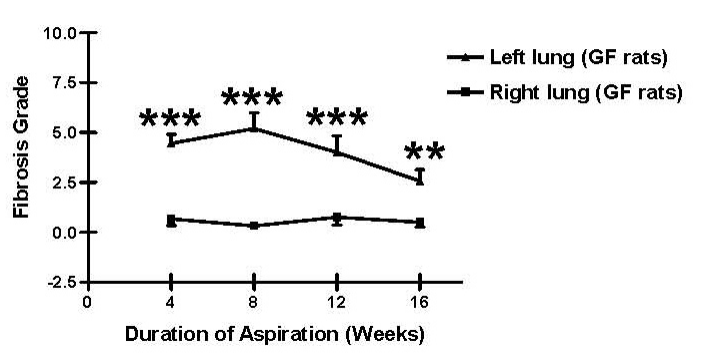
Fibrosis following gastric fluid aspiration. The fibrosis grade was evaluated as described in the Methods. The mean peribronchiolar fibrosis grade was significantly higher in left (treated) lung specimens compared to right (untreated) lung specimens after 4, 8, 12, and 16 weeks of gastric fluid aspiration. **p < 0.01, ***p < 0.001 based on two-tailed Student's t test.

Histology specimens from left lungs after 4, 8, 12, and 16 weeks of gastric fluid aspiration were compared to those from right lungs and to specimens from untreated rats and from rats receiving normal saline (Figure 2). Four weeks of gastric fluid aspiration was associated with an increase in peribronchiolar and interstitial fibrosis compared to controls (Figures 2a and 2b). These differences were greatest after 8 weeks of gastric fluid aspiration. Fibrosis was also noted after 12 or 16 weeks of gastric fluid aspiration, although it was less prominent. Based on a blinded assessment of the pathology, the fibrosis grade [17] was significantly more severe in left lung specimens compared to right lung specimens after 4, 8, 12, and 16 weeks of gastric fluid aspiration (Figure 3).

As early as 4 weeks after the initiation of gastric fluid aspiration, cellular infiltrates were observed in the left lung (Figure 2). These infiltrates tended to be most prominent after 8 weeks, diminishing but still persisting to a degree after 12 and 16 weeks of gastric fluid aspiration. Lesions consisted primarily of scattered giant cells (Figure 2c), increased peribronchiolar and perivascular lymphocytes (Figures 2a and 2d). In some instances, complete luminal obstruction of the small airways was observed (Figure 2e). Giant cell or lymphocytic infiltrates were not observed in specimens from untreated rats or from rats receiving normal saline (Figure 2b) or in the right lung of rats receiving gastric fluid (Figure 2f).

### Cellular analysis of BAL specimens

**Figure 4:**
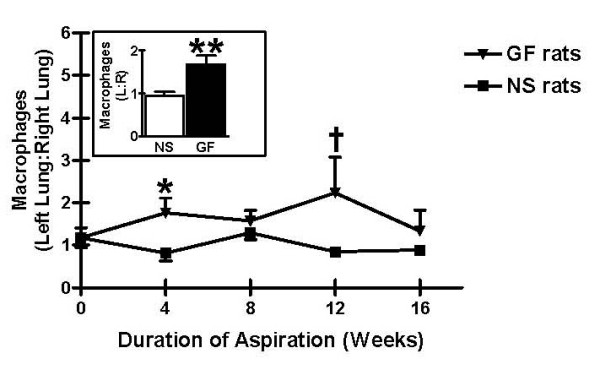
Macrophage infiltration following chronic aspiration of gastric fluid. The relative macrophage quantities in bronchoalveolar lavage (BAL) specimens following chronic aspiration of either gastric fluid or normal saline are shown. The ratio of macrophages in the left to the macrophage in the right lung is shown as a function of time. In the inset, the ratio of macrophages in the left to the macrophages in the right lung is shown for all rats, regardless of the duration of treatment. ^†^p < 0.10, *p < 0.05, **p < 0.01 based on two-tailed Student's t test.

Based on FACS analysis, the numbers of macrophages were increased in BAL specimens from left (treated) lungs of rats receiving gastric fluid, but not in BAL specimens from right (untreated) lungs of rats receiving gastric fluid, left lungs from rats receiving normal saline, or left lungs from untreated rats. Furthermore, the ratio of macrophages in the left (treated) lung to macrophages in the right (untreated) lung was significantly higher among rats receiving gastric fluid for 4 weeks compared to rats receiving normal saline for the same time period (p = 0.04). This trend was still observed after 8, 12, and 16 weeks, although the difference between experimental animals and controls was not significant (Figure 4). Among all rats receiving gastric fluid, the ratio of macrophages in the left (treated) lung to macrophages in the right (untreated) lung was significantly higher among rats receiving gastric fluid compared to rats receiving normal saline (Figure 4 inset, p < 0.01).

**Figure 5:**
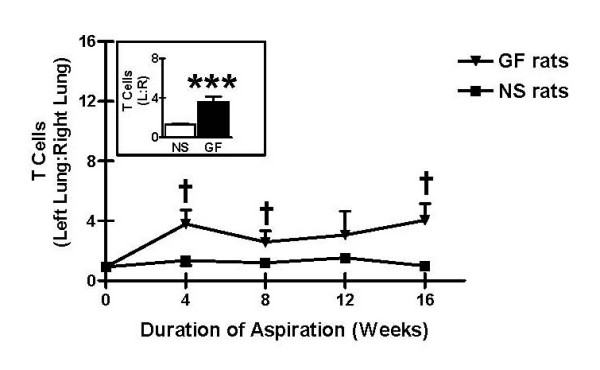
T-cell infiltrates as a result of chronic aspiration of gastric fluid. Relative T cell quantities in bronchoalveolar lavage (BAL) specimens following aspiration of either gastric fluid or normal saline are shown. Left:right lung T cell quantities were substantially higher in BAL specimens from rats receiving gastric fluid compared to rats receiving normal saline after 4, 8, 12, and 16 weeks of aspiration. ^†^p < 0.10, ***p < 0.001 based on two-tailed Student's t test.

BAL specimens from rats receiving gastric fluid exhibited a greater number of T cells compared to specimens from rats receiving normal saline after 4, 8, 12, and 16 weeks of treatment. Accordingly, the ratio of T cells in the left (treated) lung to T cells in the right (untreated) lung was consistently higher in BAL specimens from rats receiving gastric fluid compared to those from rats receiving normal saline after 4, 8, 12, or 16 weeks, although this difference was not statistically significant (Figure 5). On the other hand, when all rats receiving gastric fluid were compared to all rats receiving normal saline, regardless of the duration of treatment, the ratio of T cells in the left (treated) lung to T cells in the right (untreated) lung was significantly higher in BAL specimens from rats receiving gastric fluid compared to those from rats receiving normal saline (Figure 5 inset, p < 0.001).

**Figure 6:**
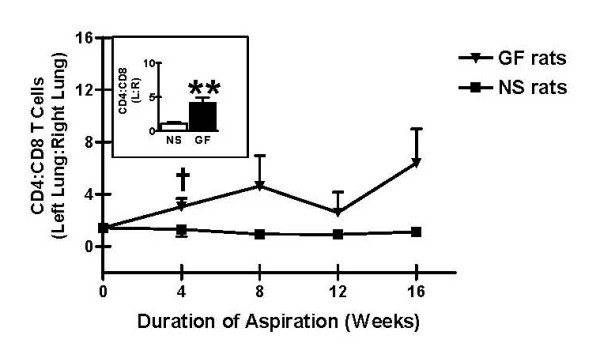
Changes in CD4:CD8 T cell ratios as a result of chronic aspiration of gastric fluid. Relative CD4:CD8 T cell ratios in bronchoalveolar lavage (BAL) specimens following aspiration of either gastric fluid or normal saline are shown. ^†^p < 0.10, **p < 0.01 based on two-tailed Student's t test.

Further analysis of BAL T cell subpopulations revealed that the CD4:CD8 T cell ratio was consistently higher in left (treated) lung specimens compared to right (untreated) lung specimens in rats receiving gastric fluid aspiration. In contrast, this increase in the CD4:CD8 T cell ratio in the left (treated) lung compared to the right (untreated) lung was not observed after normal saline aspiration (Figure 6). Using this measure, the difference between the gastric fluid and normal saline groups was not statistically significant after 4, 8, 12, or 16 weeks of treatment. However, when all rats were evaluated collectively, regardless of the duration of treatment, the relative increase in CD4:CD8 T cell ratios in the left (treated) lung compared to the right (untreated) lung was significantly greater among rats receiving gastric fluid compared to those receiving normal saline. (Figure 6 inset, p < 0.01).

### Cytokine analysis of BAL specimens

**Figure 7:**
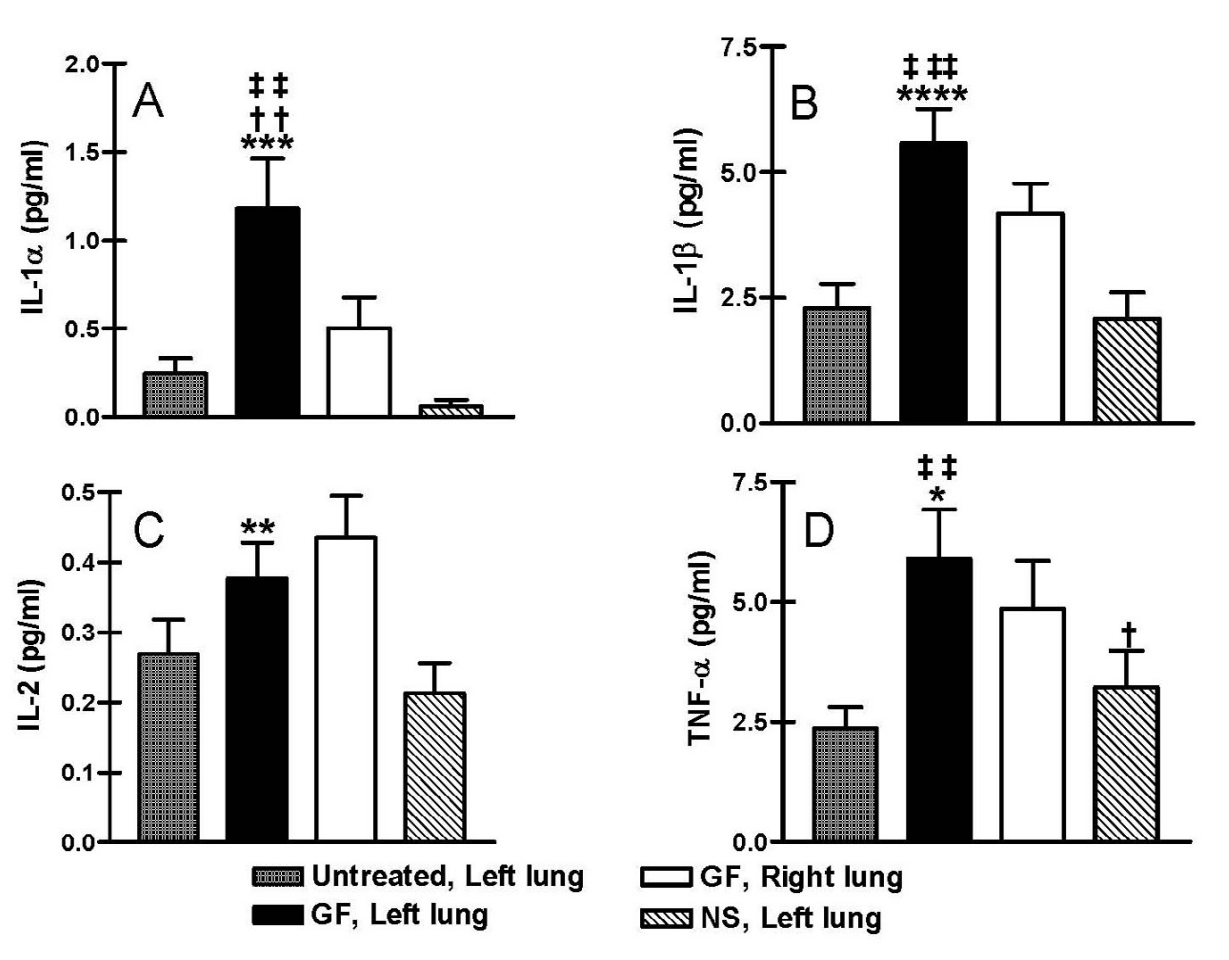
Cytokine response to chronic aspiration of gastric fluid. Cytokine levels were evaluated in the bronchoalveolar lavage (BAL) fluid from the left lung of rats receiving gastric fluid in their left lung, the left lung of rats receiving normal saline in their left lung, the right lung of rats receiving gastric fluid in their left lung, and the left lung of rats receiving no treatment. Levels of (a) IL-1alpha (b) IL-1beta, (c) IL-2 and (d) TNF-alpha are shown. ^††^p < 0.05 vs. right (untreated) lung after gastric fluid; *p < 0.10, **p < 0.05, ***p < 0.01, ****p < 0.001 vs. left (treated) lung after normal saline; ^‡‡^p < 0.05, ^‡‡‡^p < 0.01 vs. BAL from untreated rats based on two-tailed Student's t test.

Of the cytokines tested, only IL-1alpha, IL-1beta, IL-2, TNF-alpha, and TGF-beta were consistently detected in BAL specimens. Compared to untreated rats or rats receiving normal saline, IL-1alpha, IL-1beta, IL-2, and TNF-alpha were higher in BAL specimens from rats receiving gastric fluid (Figure 7). IL-1alpha was significantly higher among left (treated) lung BAL specimens from rats receiving gastric fluid compared to specimens from the right (untreated) lungs of the same animals (p < 0.05), specimens from rats receiving normal saline (p < 0.01), or specimens from untreated rats (p < 0.05) (Figure 7a). In repetitive aspiration rats, left lung specimens exhibited IL-1beta concentrations substantially higher than right lung specimens from the same rats (p = 0.12) and significantly higher than specimens from untreated rats (p < 0.01) or rats receiving normal saline (p < 0.001) (Figure 7b).

Interestingly, IL-2 and TNF-alpha were elevated to comparable levels in both left and right lung BAL specimens from rats receiving gastric fluid (Figure 7c and 7d). However, IL-2 concentrations in left lung specimens from rats receiving gastric fluid were markedly higher than those from untreated rats (p = 0.18) and rats receiving normal saline (p < 0.05). Similarly, TNF-alpha levels were also markedly higher among specimens from rats receiving gastric fluid compared to untreated rats (p < 0.05) and rats receiving normal saline (p = 0.08).

**Figure 8:**
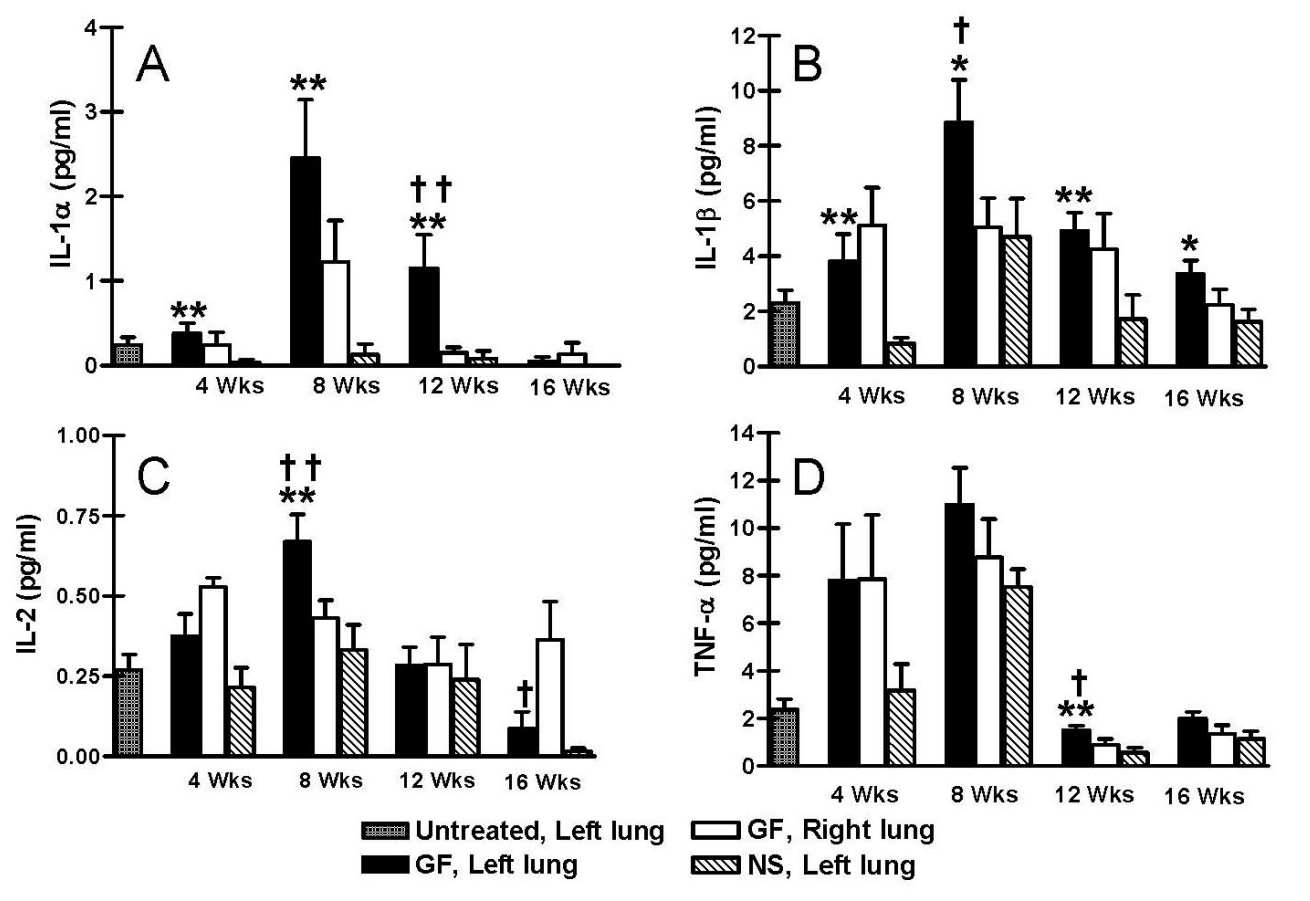
Cytokine response as a function of duration of gastric fluid aspiration. Cytokine levels in bronchoalveolar lavage (BAL) fluid are shown after 4, 8, 12 and 16 weeks of aspiration. Levels of (a) IL-1alpha, (b) IL-1beta, (c) IL-2, and (d) TNF-alpha are shown.^†^p < 0.10, ^††^p < 0.05 vs. right (untreated) lung after gastric fluid; *p < 0.10, **p < 0.05 vs. left (treated) lung after normal saline based on two-tailed Student's t test.

**Figure 9:**
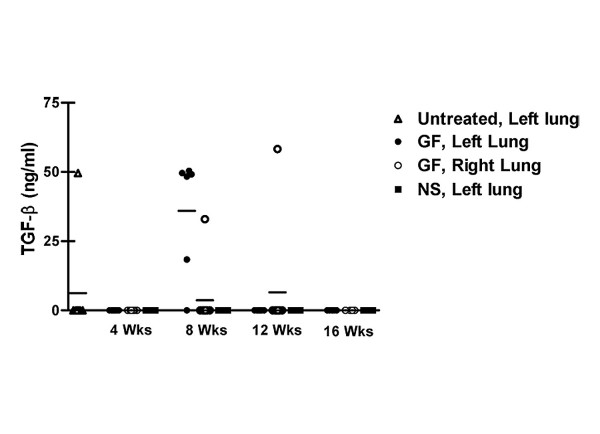
TGF-beta production in response to chronic aspiration of gastric fluid. TGF-beta was detected in BAL fluid from 5 of 6 rats after 8 weeks of gastric fluid aspiration. In contrast, TGF-beta was undetectable in almost all BAL specimens from untreated rats or from rats receiving normal saline (p < 0.0001 based on chi-square analysis).

In all instances, cytokine levels were highest in BAL specimens from rats after 8 weeks of gastric fluid aspiration (Figures 8a–d and 9). BAL levels of IL-1alpha, IL-1beta, IL-2, and TNF-alpha peaked after 8 weeks of gastric fluid aspiration and tapered off by 12 weeks and 16 weeks of gastric fluid aspiration. At most time points, BAL IL-1alpha, IL1-beta, and TNF-alpha levels were significantly higher among rats receiving gastric fluid compared to untreated rats or rats receiving normal saline (Figures 8a, b, and 8d). Furthermore, TGF-beta, which was not detectable in the majority of BAL specimens, was detected in 5 of 6 specimens from rats undergoing 8 weeks of gastric fluid aspiration (Figure 9, p < 0.0001 based on chi-square analysis).

## Discussion

The inflammatory response to massive acute aspiration events has been described previously based on animal models dating back 35 to 40 years [15,16,18]. The initial 1–2 hours are characterized by an immediate chemical burn, primarily attributed to the acidity of gastric contents, which is associated with endothelial cell damage, increased capillary permeability, and scattered intraalveolar hemorrhage. Several hours later, an acute inflammatory response follows, comprised primarily of alveolar neutrophils and macrophages. After approximately 15 hours, however, the inflammatory response resolves and pulmonary capillary permeability returns to baseline [16].

In contrast, the pathophysiologic effects of chronic aspiration are much less clear. Clinically, lung injury due to repetitive aspiration in patients with GERD has been associated with a number of pulmonary disorders including idiopathic pulmonary fibrosis, asthma, chronic bronchitis, cystic fibrosis, and chronic obstructive pulmonary disease [1-5]. Data from a number of clinical studies suggest that chronic aspiration in the context of GERD is associated with increased rates of BOS and mortality in lung transplant recipients [9-14]. However, it is unclear whether chronic aspiration in the context of GERD causes and/or exacerbates pulmonary disease or vice-versa. Furthermore, the cellular processes that contribute to such injury have not yet been characterized.

This work provides the first experimental animal model aimed at evaluating the pathogenesis of chronic aspiration-associated disease. In the current study, histology specimens from rats undergoing experimentally induced gastric fluid aspiration demonstrated increased fibrosis and a considerable giant cell infiltrate. Although the extent of fibrosis in rats undergoing aspiration of gastric fluid remained persistently greater than controls, the magnitude decreased slightly with prolonged aspiration, the relevance of which is unclear from these studies. Additionally, perivascular and peribronchial lymphocytic infiltrates were seen, as well as fibroproliferative lesions obstructing terminal airways, histologic findings comparable to those of acute and chronic rejection, respectively, in lung transplant recipients. Although these lesions were most prominent in rats following 8 weeks of gastric fluid aspiration, they were evident at all time points. Interestingly, these lesions were similar to the scattered granulomatous response and obstructive bronchiolitis pattern described by Teabeaut that occurred in some instances following an acute aspiration event in rabbits [18].

Analysis of BAL specimens revealed a substantial increase in macrophages and T cells (particularly after 4 weeks of gastric fluid aspiration) that persisted throughout the study period. Notably, the increase in T cells was characterized by a prominent shift toward a higher CD4:CD8 T cell ratio, which some authors have previously correlated with OB (obliterans bronchiolitis) in lung transplant recipients [19,20]. Furthermore, repetitive gastric fluid aspiration also resulted in increased TGF-beta, TNF-alpha, IL-1alpha, IL-1beta, and IL-2 concentrations in BAL specimens compared to controls, a TH1 cytokine-dominated profile, whereas no increase in TH2 cytokines, such as IL-4, IL-6, and IL-10, was detected.

One explanation for these observations is that repetitive aspiration events may result in an early macrophage response that generates TGF-beta, TNF-alpha, IL-1alpha, and IL-1beta. Chemotactic for fibroblasts, TGF-beta induces fibrosis and remodeling of the extracellular matrix [21]. Production of TNF-alpha stimulates a generalized inflammatory response, not only potentiating fibrosis, but also inducing the upregulation of adhesion molecules and the production of additional cytokines, including IL-1alpha and IL-1beta. TNF-alpha also plays a critical role in leukocyte trafficking and homing of T and B cells [22,23]. The synthesis of IL-1alpha and IL-1beta by endothelial cells, fibroblasts, and macrophages can be profibrotic and not only exacerbates the inflammatory response, but also activates and stimulates proliferation of T and B cells [21]. Activated T cells produce additional IL-1alpha and IL-1beta as well as IL-2, which further propagate the inflammatory response by activating macrophages, natural killer cells, and lymphokine-activated killer cells. Furthermore, these cytokines stimulate differentiation and proliferation of T and B lymphocytes thereby directing or upregulating the cell-mediated and humoral immune responses. Such an upregulated immune response may have substantial effects on pulmonary pathology beyond mediation of lung transplant rejection. Since the lung is exposed constantly to numerous environmental antigens, altered immunoreactivity against these antigens may have a substantial effect on normal lung function.

By exacerbating the pulmonary immune response, it is possible that such an inflammatory milieu could initiate the development of various pulmonary diseases. An individual's response may influence the phenotypic response in the lung – the majority of patients may exhibit a normal reparative response whereas certain individuals may be more prone to fibrosis or other pathophysiology. For instance, such altered function may play a role in the asthmatic response, which is thought to depend primarily upon the interaction of mast cells, eosinophils, macrophages, CD4+ T cells, and IgE-producing B cells [24-26]. It is possible that the presence of macrophages and CD4+ T cells in patients with chronic aspiration may lower the threshold for an immune-mediated asthmatic response by inducing isotype-switching to IgE production in B cells [24].

It is also quite possible that, in the setting of a pulmonary allograft, the development of these inflammatory mediators could recruit and/or exacerbate immune responses that predispose recipients to acute and/or chronic rejection. The activation of TH1 immune pathways and the generation of a cytotoxic response has been associated with rejection in a number of lung allograft models [27,28]. A principal promoter of T cell activation and cytotoxic function, IL-2 is commonly detected in recipients of lung and other allografts during OB and/or rejection [29-32]. Additionally, TGF-beta has been associated with the tissue remodeling response that occurs during the development of OB and has been used as an early marker for the process [33-35]. TNF-alpha increases class I MHC expression and has been associated with acute and chronic rejection in recipients of lung and other allografts [21,36-38]. Furthermore, blocking TGF-beta, TNF-alpha, or IL-1 prevents airway matrix deposition and OB in animal models [38-40].

In our study, it appears that the native lung eventually develops tolerance to the injury induction by chronic aspiration. Peribronchiolar and interstitial fibrosis, as well as cellular infiltrates began at 4 weeks, peaked at 8 weeks, and then appeared to regress after that time. BAL specimens showed that macrophage infiltrates appeared to peak by 8 weeks, and that cytokine levels peaked at 8 weeks. The immune responses initially induced by repetitive aspiration events thus build over the first 8 weeks with corresponding worsening of the histopathologic appearance of the involved lung. As cytokine concentrations and inflammatory cell populations then diminish, presumably via mechanisms of immunologic tolerance and/or protective structural changes in the lung, the degree of fibrosis and airway pathology begin to normalize. It is possible that this improvement would eventually plateau at some level of permanent fibrosis and inflammatory activation above the initial baseline, or that improvement would continue to resolution given enough time.

These preliminary studies have several inherent limitations. First, weekly administration of gastric fluid to rats may not be representative of clinical GERD, which often affects patients at more frequent intervals. For this reason, more physiologic studies involving GERD induction in rats are underway in our laboratory. This study does not address what component of gastric fluid is primarily responsible for the observed pathologic changes. Normal saline controls also reveal that gastric fluid is not necessary to induce elevation of TNF-alpha levels (see figure 7). This could be due to mechanical effects of even physiologically inert fluid in air spaces, or to repetitive anesthesia, intubation, and mechanical ventilation. Vaneker et. al. recently showed in a rodent model that mechanical ventilation with clinically relevant ventilator settings caused reversible increases in immune cytokine concentrations (including TNF-alpha) and leukocyte influx in lung tissue [41]. The absence of significant histologic changes seen in their study also agrees with our findings.

Nevertheless, this work is expected to provide a basis for future studies. Foremost among those studies will be the evaluation of the components of gastric fluid that are primarily responsible for the inflammatory response resulting from chronic aspiration. Major components of gastric fluid include hydrogen, potassium, sodium, and chloride ions, pepsin, bile salts, and food particles. Although the concentrations of these different components are highly variable depending on the animal's time since feeding, diet, age, state of stress, and numerous other variables, approximate values have been reported in the literature: pH 1.0 – 3.5, Sodium 50 mEq/l, Potassium 9.0 mEq/L, Chloride 135 mEq/l, Pepsin 0.5 mmol/L (or 2300 – 3100 U/mL), Bile Salts 0.05 – 0.15 mmol/L [42-45]. Given its known role in acute aspiration, the acidity of gastric fluid might be expected to be a major etiologic factor in the injury seen in this model in which gastric fluid pH ranged between1.0 – 2.5. Based on data from our institution, however, it seems unlikely that the acidic component of the gastric contents is solely responsible for poor outcomes, since the administration of H2 blockers or proton pump inhibitors to lung transplant recipients with GERD does not prevent their clinical deterioration [9,11,13,14]. Other components may thus play an even more important role. For instance, lipopolysaccharide, commonly present in gastric secretions, can induce a neutrophilic alveolitis [46], and a high concentration of bile acids in post lung transplant BAL samples were associated with earlier onset of BOS [47]. Determination of the components of gastric fluid that are primarily responsible for the observed pathology may facilitate the development of pharmacologic interventions aimed at the pathologic processes associated with chronic GERD.

## Conclusion

Clinical data suggests that chronic aspiration contributes to pulmonary injury, resulting in a variety of pulmonary pathologies. Based on the rodent model of chronic aspiration described herein, chronic aspiration can initiate an inflammatory response consisting of macrophages and T cells and characterized by increased TGF-beta, TNF-alpha, IL-1alpha, IL-1beta, IL-2 and fibrosis in the lung. The increased production of TGF-beta and TH1 cytokines following repetitive aspiration events further suggests that chronic aspiration augments pulmonary injury. These observations provide further support for the role for chronic aspiration in the development of pulmonary fibrosis, OB, and asthma. This work also provides a springboard for future studies aimed at better characterization of the pathways and effector molecules involved in chronic aspiration-associated pulmonary dysfunction.

## Competing interests

The author(s) declare that they have no competing interests.

## Authors' contributions

JA carried out gastric fluid aspirations, assisted with analysis of cytokine levels, and histology data, and helped to prepare the manuscript. SML helped with analysis of cytokine data and assisted with manuscript preparation. MH helped perform the gastric fluid aspirations and with data analysis. BL carried out the cytokine analysis, and helped prepare figures for the manuscript. CH assisted with animal care and organ preparation for histologic examination, assisted with histology data analysis. EC helped to design the project and carried out preliminary experiments validating aspiration technique. YY assisted in developing the methodology of gastric fluid collection and aspiration. SSL obtained funding for this project, assisted with experimental design, and performed histologic analysis. WP assisted with the experimental design, and with manuscript preparation. RDD obtained funding for this project and assisted with project conception and design.
